# Radiomics for differentiation of somatic *BAP1* mutation on CT scans of patients with pleural mesothelioma

**DOI:** 10.1117/1.JMI.11.6.064501

**Published:** 2024-12-11

**Authors:** Mena Shenouda, Abbas Shaikh, Ilana Deutsch, Owen Mitchell, Hedy L. Kindler, Samuel G. Armato

**Affiliations:** a The University of Chicago, Department of Radiology, Chicago, Illinois, United States; b Rice University, Houston, Texas, United States; c Northwestern University, Evanston, Illinois, United States; d The University of Chicago, Department of Medicine, Chicago, Illinois, United States

**Keywords:** BRCA1-associated protein 1, mesothelioma, classification, somatic mutation, deep learning, U-Net, computed tomography scan

## Abstract

**Purpose:**

The BRCA1-associated protein 1 (*BAP1*) gene is of great interest because somatic (*BAP1*) mutations are the most common alteration associated with pleural mesothelioma (PM). Further, germline mutation of the *BAP1* gene has been linked to the development of PM. This study aimed to explore the potential of radiomics on computed tomography scans to identify somatic *BAP1* gene mutations and assess the feasibility of radiomics in future research in identifying germline mutations.

**Approach:**

A cohort of 149 patients with PM and known somatic *BAP1* mutation status was collected, and a previously published deep learning model was used to first automatically segment the tumor, followed by radiologist modifications. Image preprocessing was performed, and texture features were extracted from the segmented tumor regions. The top features were selected and used to train 18 separate machine learning models using leave-one-out cross-validation (LOOCV). The performance of the models in distinguishing between *BAP1*-mutated (*BAP1+*) and *BAP1* wild-type (*BAP1−*) tumors was evaluated using the receiver operating characteristic area under the curve (ROC AUC).

**Results:**

A decision tree classifier achieved the highest overall AUC value of 0.69 (95% confidence interval: 0.60 and 0.77). The features selected most frequently through the LOOCV were all second-order (gray-level co-occurrence or gray-level size zone matrices) and were extracted from images with an applied transformation.

**Conclusions:**

This proof-of-concept work demonstrated the potential of radiomics to differentiate among *BAP1+/−* in patients with PM. Future work will extend these methods to the assessment of germline *BAP1* mutation status through image analysis for improved patient prognostication.

## 1 Introduction

The use of radiomics, specifically texture analysis, has long been implemented in medicine to help clinicians and researchers extract quantitative information from images.^1^^–^^4^ Advances in the field have linked imaging features with patients’ genetic profiles, i.e., “imaging genomics.”^5^^,^^6^ Imaging genomics has been applied to many different diseases and anatomic regions.^7^ For example, Velazquez et al.^8^ were able to discriminate between cases with and without a somatic mutation in the epidermal growth factor receptor gene using radiomic signatures acquired from computed tomography (CT) scans of adenocarcinoma patients. Similarly, Yip et al.^9^ performed the same task using positron emission tomography images of patients presenting with non-small cell lung cancer.

The use of imaging genomics for pleural mesothelioma (PM) is rare in the literature. PM is an aggressive form of cancer present in the pleural lining of the lung, resulting from exposure to asbestos, and has a very poor prognosis. The BRCA1-associated protein-1 (*BAP1*) gene encodes for the BAP1 protein, a deubiquitinase that influences cell growth, cell proliferation, and cell death.^10^^–^^12^ It is of great interest because it accounts for the most common somatic mutations in PM.^12^^,^^13^ *BAP1* mutations can also be inherited, and individuals with germline mutations in this gene have been widely recognized as being predisposed to the disease, although patients with a germline *BAP1* mutation are associated with better prognosis^13^^,^^14^ than those without the germline mutation, with a sevenfold increase in long-term survival regardless of sex and age.^15^ By identifying suspected germline mutations solely through radiomics, clinicians could be prompted to pursue genetic testing, which is not currently the standard of care,^16^ resulting in more streamlined patient prognostication and assessment of family members, who have a 50% chance to inherit the same mutation.^14^ To determine the feasibility of future studies in identifying the germline mutation status from medical images, this novel work^17^ first explored the use of radiomics on the CT scans of PM patients with the more prevalent somatic *BAP1* mutations.^15^^,^^18^^–^^20^

## 2 Methods

**Fig. 1:**
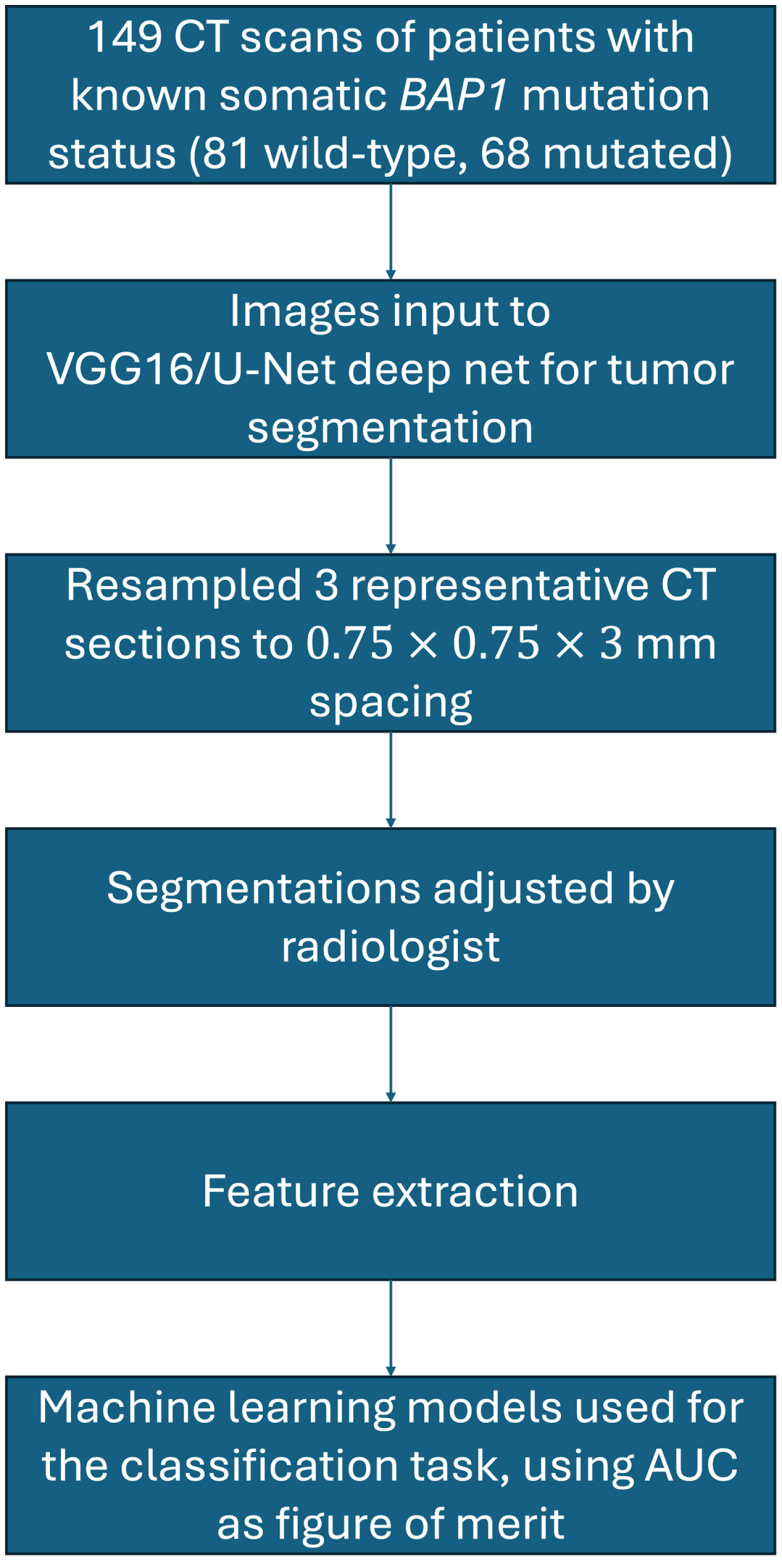
Pipeline incorporated in this study, beginning with the patient cohort curated and ending with the machine learning models used for the *BAP1* classification task.

The overall workflow for this work is presented in Fig. 1.

### 2.1 Patient Selection and Sample Collection

This study curated 149 patients diagnosed with PM from the University of Chicago Medicine (UCM) under a Health Insurance Portability and Accountability Act–compliant, Institutional Review Board–approved protocol from April 2016 to June 2022. Informed consent was obtained from all participants. The protocol allowed for the collection and biobanking of peripheral blood, saliva, and tumor samples. Tumor deoxyribonucleic acid was extracted from fresh frozen, paraffin-embedded tumor tissue blocks. Somatic mutations were identified using the UCM OncoPlus next-generation sequencing panel.^21^ Patients with confirmed somatic *BAP1* mutations only (*BAP1+*, N=68) were included in the study. The remaining 81 patients presented with the wild-type allele (*BAP1−*). Immunohistochemical analysis of the BAP1 protein was conducted in a Clinical Laboratory Improvement Amendments–certified laboratory at UCM using the Santa Cruz C4 monoclonal antibody. Table 1 includes further details about the patients of this study.

**Table 1:** Patient demographics categorizing patient sex and age characteristics.

	Total (n=149)	*BAP1+* (n=68)	*BAP1−* (n=81)
Sex	—	—	—
Male	95	48	47
Female	54	20	34
Age	—	—	—
Median	69	69.5	69
Range	21 to 90	51 to 90	21 to 81

### 2.2 Image Data Curation and Segmentation

Axial images from unenhanced chest CT scans of the patients were retrospectively collected (Table 2).^22^ Scans were acquired with the assistance of the University of Chicago’s Human Imaging Research Office,^23^^,^^24^ which provided de-identified, compliant images for evaluation. For each patient, the CT section displaying the largest area of tumor was selected by a radiologist. This section and the immediate superior and inferior sections were used to create a three-dimensional (3D) volume for analysis. A Visual Geometry Group 16 (VGG16)/U-Net deep convolutional neural network (CNN) architecture was utilized to segment the tumor within this volume.^25^ The two-dimensional (2D) architecture employed downsampling and upsampling paths. The downsampling path utilized a VGG16 model pre-trained on ImageNet with scale-jittering, applying 2×2 max pooling with stride 2. Dropout layers of 0.5 probability were used to prevent model overfitting. The upsampling path employed a 2D operation with nearest-neighbor interpolation on the feature maps. The network generated 512×512-pixel probability maps, which matched the input image size. Rectified linear unit and sigmoid activation functions were applied after the convolutional layers and the final layer, respectively. Lastly, the model was trained with a binary cross-entropy loss function using the Adam optimizer with a learning rate of $10^{-5}$. More details regarding the architecture of the model and its training scheme can be found in Gudmundsson et al.^25^ For the present study, tumor contours were automatically generated and evaluated with no additional training or validation of the model.

**Table 2:** Image acquisition characteristics for the patient cohort analyzed in this study.

	Total (n=149)	*BAP1+* (n=68)	*BAP1−* (n=81)
Pixel size (mm)	—	—	—
Median	0.72	0.71	0.73
Range	0.56 to 1.07	0.57 to 1.07	0.56 to 0.95
Slice thickness (mm)	—	—	—
Median	3	2.5	3
Range	1 to 5	1 to 5	1 to 5
kVp (kV)	—	—	—
Median	120	120	120
Range	80 to 140	100 to 120	80 to 140
Scanner manufacturer	—	—	—
GE	73	35	38
Philips	45	21	24
Toshiba	13	5	8
Siemens	18	7	11
Reconstruction kernel	—	—	—
GE: standard	71	34	37
GE: chest	2	1	1
Philips: B	44	21	23
Philips: C	1	0	1
Toshiba: FC13	6	1	5
Toshiba: FC14	2	1	1
Toshiba: FC18	5	3	2
Siemens: B30f	3	0	3
Siemens: B31f	1	0	1
Siemens: B40f	1	0	1
Siemens: B31s	1	0	1
Siemens: B35s	1	1	0
Siemens: Bf39f	2	1	1
Siemens: Bf37f	1	0	1
Siemens: Br36f	1	0	1
Siemens: Br40d	1	1	0
Siemens: I26f	1	0	1
Siemens: I31f	4	3	1
Siemens: I41f	1	1	0

The resulting probability maps output by the network were thresholded at a value of 0.2; this threshold was determined to have maximal overlap with human contours using the Dice similarity coefficient (DSC) from prior work.^26^^,^^27^ The radiologist adjusted the resulting segmentations to ensure the segmentations were highly specific to tumor pixels. The finalized contours were defined as the region of interest (ROI) and used for feature extraction.

### 2.3 Image Resampling and Gray-Level Discretization

**Fig. 2:**
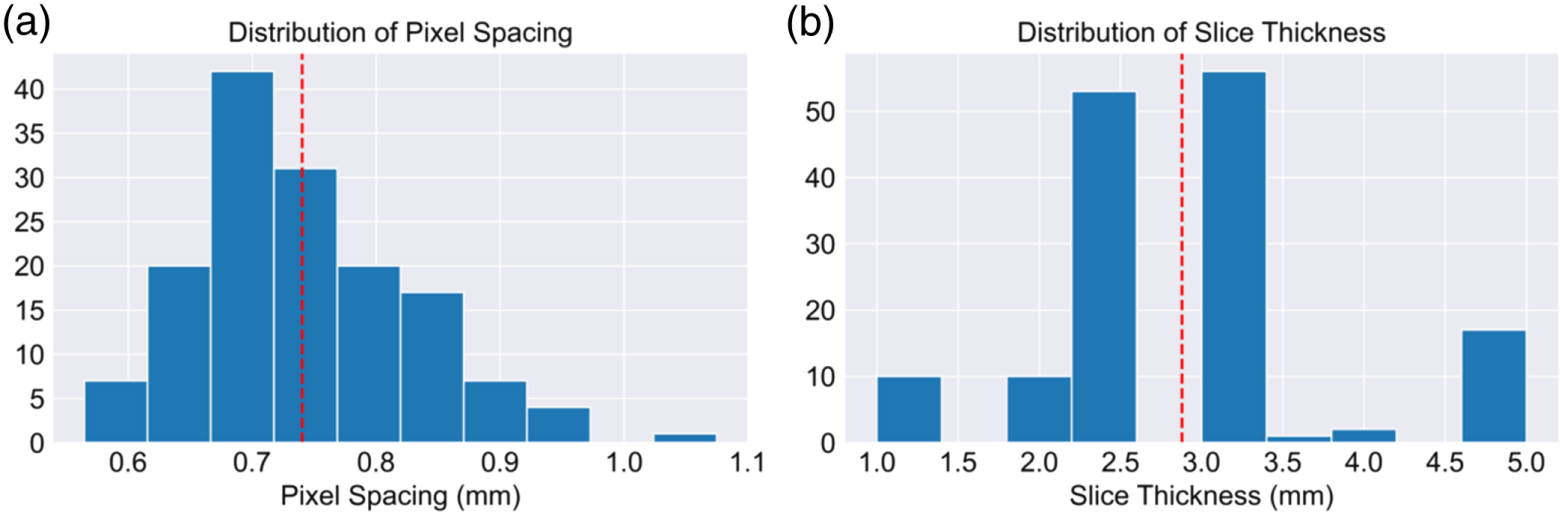
Histogram of the (a) pixel spacing and (b) slice thickness of CT sections of the original 149 scans. The red vertical line depicts the mean value in each of the distributions to which resampling was performed.

To mitigate the impact of different image acquisition parameters, all images were resampled to the mean resolution of all scans, with a pixel spacing of 0.75×0.75  mm and a slice thickness of 3 mm (see Table 2 and Fig. 2 for more details). Prior to texture feature extraction, gray-level discretization was applied using a fixed bin number of 32 gray levels, as small or large gray-level quantizations have been shown to impact texture feature values due to the reduction of information that can be extracted from an image.^28^^,^^29^

### 2.4 Feature Extraction

Eighteen intensity-based and 123 texture features (111 second-order, 6 Laws’ texture energy, 2 Fourier, and 4 fractal dimensions) were extracted from the original ROIs. The 123 texture features were also extracted from the ROIs after applying seven different filtering operations on the images: two Laplacian of Gaussian (LoG) filters (σ=0.75  mm, 1.5 mm), four multi-channel wavelet decompositions [low low (LL), low high (LH), high low (HL), and high high (HH)], and a local binary pattern operator (radius = 0.75 mm). With 18 intensity-based features and 123 texture features extracted from the ROIs before the filtering operations and the 123 features extracted from the ROIs after the seven filtering operations, a total of 1002 features were computed from each ROI (the finalized tumor contours). Intensity-based features were obtained from the 3D volume.^30^ All other features were computed by averaging the 2D feature values over the three CT sections. Features were calculated using the Python packages PyRadiomics,^31^ PyFeats, and Nyxus.

### 2.5 Data Imbalance

Due to the imbalance of *BAP1* mutation status among patients, a hybrid approach using the Synthetic Minority Over-sampling Technique (SMOTE) coupled with the removal of Tomek links was employed to over-sample the minority class and under-sample all classes,^32^ respectively, prior to the feature selection. The SMOTE algorithm generates artificial data in the feature space near the existing feature values of cases from the minority class. Tomek links are a pair of nearest neighbors of opposite classes with minimal distance between them compared with other neighboring data. Removal of Tomek links decreases noisy data or eliminates data near the decision boundary. Implementation of SMOTE–Tomek resulted in equal mutation prevalence, per fold, during training.

### 2.6 Machine Learning Model and Feature Selection

The performance of 18 separate^33^ calibrated machine learning models (Table 3) was evaluated using leave-one-out cross-validation (LOOCV), resulting in 149 iterations. Calibration was performed using the “sigmoid” method, which corresponds to fitting a logistic regression model to the scores of a classifier (Platt’s scaling). Although “isotonic” calibration, which fits a non-parametric isotonic regressor, could be performed, such calibration is recommended only for large datasets as overfitting could result in too few samples (i.e., fewer than 1000 cases).^34^^,^^35^

**Table 3:** Types of models evaluated in the *BAP1* classification task.

Linear
Logistic regression
Ridge
Stochastic gradient descent (SGD)
Passive aggressive
Neighbor
*K* neighbors
Tree
Decision tree
Extra tree
Support vector machine (SVM)
Linear SVM
Radial basis function SVM
Naive Bayes
Gaussian naive Bayes
Ensemble
AdaBoost
Bagging
Random forest
Extra trees
Gradient boosting
Gaussian process
Gaussian process
Discriminant analysis (DA)
Linear (LDA)
Quadratic (QDA)

Feature selection was performed on the training set of each iteration of the LOOCV in the following order (with empirically determined parameters):^36^ (1) features with variance less than 0.01 were discarded, (2) features were *Z*-score normalized, and (3) features with a Pearson correlation coefficient of 0.75 or greater with other features were removed (to assess linear independence among the features).^37^ Lastly, the top four features were selected using the calculated *F*-statistic of the analysis of variance (ANOVA) test between the feature and the *BAP1* mutation status.^38^ These four features were then extracted from the left-out test case, per iteration, for the classification task.

Other training schemes were assessed. In particular, different-sized folds for repeated *k*-fold cross-validation were implemented as well as changing the number of top features selected.^38^ Preliminary work was also performed to study the impact random state seeds had on the classification task.

### 2.7 Evaluation Metric and Statistical Analysis

The receiver operating characteristic area under the curve (ROC AUC) was used as the figure of merit to assess the classification performances of the models to differentiate among *BAP1+/−* patients. The Wilcoxon rank-sum test was used to assess the differences in tumor volume, and age distributions between patients in the two classes and the DeLong^39^ and Wilcoxon signed-rank tests were used to evaluate the differences in AUC values among the models. To assess the impact of human modifications on the segmentation of the PM tumor, DSC values were calculated between the CNN segmentations and radiologist-modified masks to determine the overlap between the two. Further, the classification task was performed employing the same models (Table 3) and using feature values extracted from the unmodified CNN probability maps thresholded at 0.2. Using the DeLong test, the AUC values computed from the unmodified segmentations were compared with the AUC values achieved from the modified segmentations. Due to the hypothesis-generating nature of this work, statistical significance was obtained at p=0.05.

## 3 Results

### 3.1 Tumor Volume

**Fig. 3:**
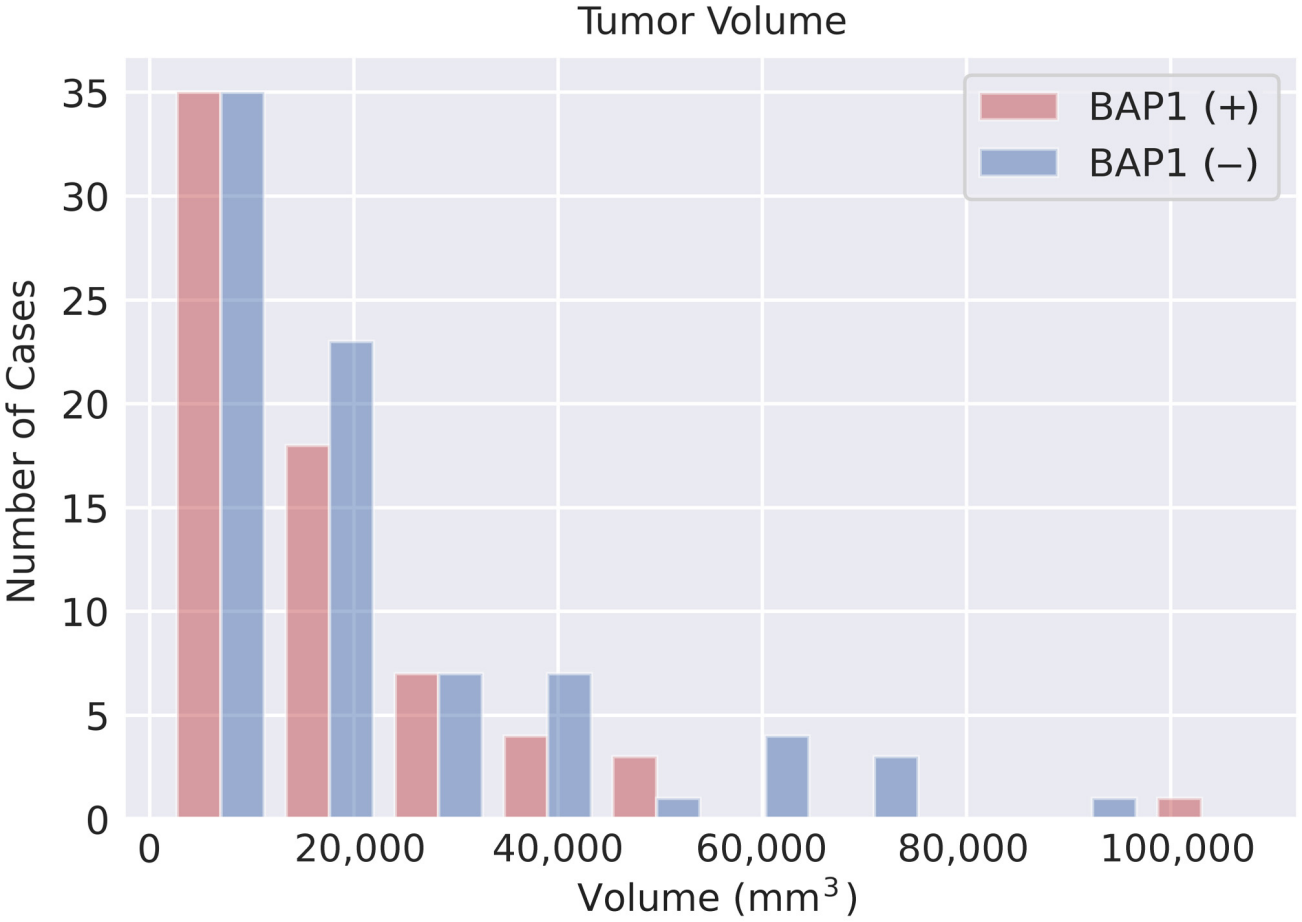
Histogram of the tumor volume categorized by *BAP1* mutation status. The difference in tumor volume between wild-type and mutated tumors failed to achieve statistical significance.

Figure 3 shows the distributions of the tumor volume contoured across the three sections selected per patient in the dataset; the median (range) volume of tumor contoured was $13,109\;{\mathrm{mm}}^{3}$ (1630 to $108,331\;{\mathrm{mm}}^{3}$) across all patients, $11,615\;{\mathrm{mm}}^{3}$ (1630 to $108,331\;{\mathrm{mm}}^{3}$) for *BAP1+* patients, and $15,949\;{\mathrm{mm}}^{3}$ (1688 to $92,352\;{\mathrm{mm}}^{3}$) for *BAP1−* patients. The difference in median volume among the *BAP1+/−* patients failed to achieve statistical significance (p=0.15), which mitigated the impact of tumor size as a confounding factor for the classification task. *BAP1+* patients had the larger range of tumor volumes, whereas *BAP1−* patients had the larger median. Differences in age among the patient cohorts failed to achieve statistical significance.

### 3.2 Classification Performance

**Fig. 4:**
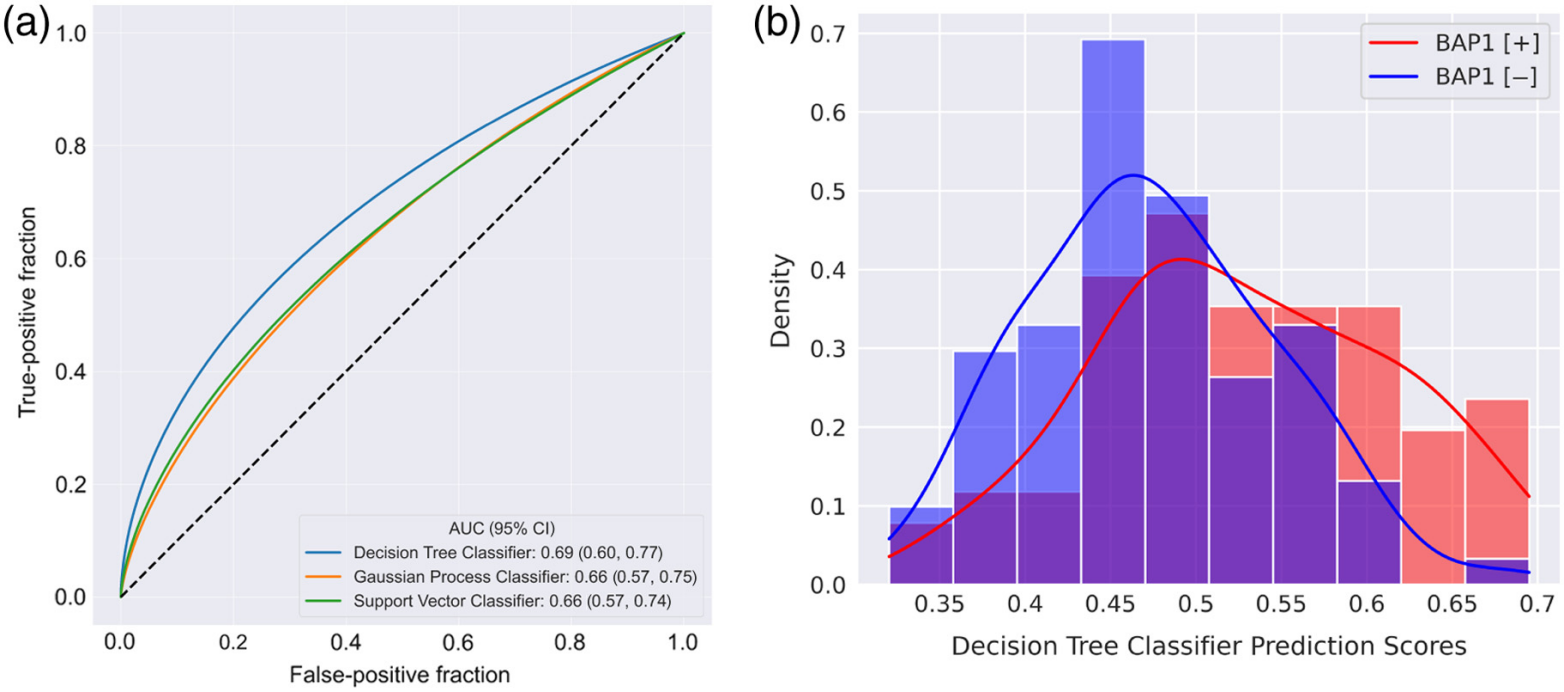
(a) ROC curves depicting the true-positive and false-positive fractions of the top three performing classifiers in the task of differentiating somatic *BAP1* mutation status using feature values extracted from segmented regions. ROC curves were fitted using software created by Metz and Pan.^40^ (b) Distributions of the decision tree classifier prediction scores across all cases. The histograms were normalized to have an equal area of 1.

In the task of differentiating between *BAP1+* and *BAP1−* patients, the top three models (sorted by AUC values) were decision tree, Gaussian process, and SVM with a radial basis function kernel (Table 3). Figure 4(a) shows the ROC curves obtained from the three models, along with their AUC values and the 95% confidence intervals (CIs). The AUC values and 95% CIs were constructed from 2000 bootstrapped samples of the prediction values during LOOCV. The decision tree classifier yielded an AUC value of 0.69 (95% CI: 0.60, 0.77). Figure 4(b) displays the distribution of scores obtained during the cross-validation for the top-performing model, the decision tree. No scores were less than 0.32 or greater than 0.70 for either class. The DeLong test failed to achieve a statistically significant difference in AUC values among the top three models as shown in Table 4.

**Table 4:** Comparisons of the three best-performing classification models: decision tree, Gaussian process, and support vector. The p-values comparing the differences in AUC values were calculated using the DeLong test, with their corresponding CIs. Significance levels (α) and widths of the CIs were adjusted for multiple comparisons. None of the comparisons achieved statistical significance after correcting for multiple comparisons using Bonferroni–Holm corrections.

Comparison	p-value for ΔAUC	α	CI of ΔAUC
Decision tree versus Gaussian process	0.4574	0.025	97.5% CI: [−0.060, 0.12]
Decision tree versus support vector	0.3208	0.017	98.3% CI: [−0.051, 0.12]
Gaussian process versus support vector	0.6478	0.050	95.0% CI: [−0.022, 0.036]

The four features selected most frequently through the 149 iterations of the cross-validation are presented in Table 5. All the features were second-order [e.g., gray-level co-occurrence (GLCM) or gray-level size zone matrices (GLSZM)] and were extracted from LoG-filtered or wavelet-decomposed images.

**Table 5:** Four texture features most often selected during the 149 LOOCV iterations and the frequency each feature was chosen, i.e., the number of iterations in which a feature was selected.

Transformation	Class	Feature	Selection frequency
LoG (σ=1.5 mm)	GLCM	Cluster prominence	149
LoG (σ=0.75 mm)	GLSZM	High gray-level zone emphasis	141
Wavelet (bior1.1–LH)	GLSZM	High gray-level non-uniformity normalized	87
LoG (σ=0.75 mm)	GLCM	Correlation	70

### 3.3 Change of *k*-Fold and Number of Features

Table 6 displays the AUC values achieved from the different number of folds used for the repeated *k*-fold cross-validation and the different number of features selected by the final ANOVA feature selection step: 200 repetitions were performed to ensure robust statistics for the calculation of the 95% CI. As reported in Sec. 3.2, the decision tree classifier resulted in the highest overall AUC value of 0.69 [0.60, 0.77]; however, this AUC value failed to achieve a significant difference (p=0.1) from the AUC value of the SGD classifier (0.63 [0.54, 0.72]) obtained when selecting the top 10 features, as determined using the DeLong test for correlated ROC comparison and setting the alternative hypothesis to “greater.”

**Table 6:** Model performance using various cross-validation approaches. ROC AUC values in the task of differentiating between *BAP1+* and *BAP1−* patients and 95% CIs for the LOOCV were obtained using 2000 bootstrapped samples. For the 10-fold and 5-fold cross-validation, AUC values were acquired by averaging the AUC values per repeat of the cross-validation approach, and 95% CIs were obtained by calculating the 2.5% and 97.5% percentile of the distribution of AUC values.

	Top model	AUC value (95% CI)	Most selected feature
200-repeat, 10-folds	—	—	—
Selecting the top 4 features	Extra trees classifier	0.58 [0.52, 0.67]^b^^,^^d^	LoG_sigma = 2.0 GLCM
			Cluster prominence
Selecting the top 10 features	Gaussian naive Bayes	0.58 [0.53, 0.62]^b^^,^^e^	LoG_sigma = 2.0 GLCM
			Cluster prominence
200-repeat, 5-folds	—	—	—
Selecting the top 4 features	Quadratic discriminant analysis	0.57 [0.50, 0.64]^c^^,^^d^	LoG_sigma = 2.0 GLCM
			Cluster prominence
Selecting the top 10 features	*K* neighbors classifier	0.58 [0.51, 0.65]^c^^,^^e^	LoG_sigma = 2.0 GLCM
			Cluster prominence
LOOCV	—	—	—
Selecting the top 4 features	Decision tree classifier	0.69 [0.60, 0.77]^f^	LoG_sigma = 2.0 GLCM
			Cluster prominence
Selecting the top 10 features	SGD classifier	0.63 [0.54, 0.72]^f^	^a^LoG_sigma = 2.0 GLCM
			Cluster prominence

a Five other features were selected during all 149 iterations.

b Comparison is significantly different, p<0.05.

c Comparison is significantly different, p<0.05.

d Comparison is significantly different, p<0.05.

e Comparison yielded p=0.14.

f Comparison yielded p=0.1.

A selection of four features yielded a different distribution of AUC values than the distribution of AUC values calculated with a selection of 10 features (p<0.05 as determined by the Wilcoxon signed-rank test). There was a significant difference between 10- and 5-fold cross-validation results when selecting four features (p<0.05); however, this trend did not occur for a selection of 10 features as there was a failure to achieve significance (p=0.14). Interestingly, the most-selected feature was the same across all cross-validation approaches: GLCM cluster prominence with an LoG filter applied of size σ=1.5 mm (LoG_sigma = 2.0). The top-performing models encompassed different types, including ensemble, naive Bayes, discriminant analysis, neighbor, tree, and linear. Therefore, the classification schemes included all but the SVMs and Gaussian processes.

To assess the impact of the random state seed on the performance of a model, AUC values were recorded for 100 seeds of the decision tree classifier, resulting in a median AUC value of 0.66 [0.64, 0.68], with the 95% CI calculated using the percentiles for 2.5% and 97.5% of the distribution of the 100 AUC values calculated; the reported value of 0.69 obtained during LOOCV of the decision tree classifier was outside these boundaries of the CI constructed from the AUC values calculated for the 100 random seeds.

### 3.4 DSC and Classification Performance of Unmodified Segmentations

When comparing the CNN segmentations to the radiologist-modified segmentations, an average DSC value of 0.79 with an interquartile range of 0.21 was achieved. The same feature extraction and selection were performed on the unmodified segmentations of tumor contours. The CNN failed to predict tumors for one case; therefore, that case was discarded from the analysis. Using LOOCV, the highest AUC value achieved across the 18 models was 0.61. The decision tree classifier, the highest-performing model as aforementioned, yielded an AUC value of 0.45 (0.36, 0.56), which was significantly different than 0.69 (p<0.001) as determined by the DeLong test.

## 4 Discussion

This proof-of-concept work explored the feasibility of differentiating between the mutation status of somatic *BAP1* patients based solely on the 2D radiomics features extracted from patients’ CT scans. The approach in this study yielded a higher AUC value than currently reported in the literature (0.65).^41^ To the best of our knowledge, Xie et al.^41^ is the only other publication discussing *BAP1* differentiation using image analysis for mesothelioma; however, the work presented here is novel as it is the first to synergistically implement a deep learning model for tumor segmentation and machine learning models for *BAP1* classification.

Prior to the feature extraction, there was careful consideration in the selection of the “standard” reconstruction for all patient scans in an attempt to choose this reconstruction across the different scanner manufacturers and kernel nomenclature. In addition, differences in pixel and axial dimension spacing due to the variability of image acquisitions from different institutions and different scanners were mitigated by image resampling, as resampling prior to feature extraction has been shown to decrease the variability of radiomic features.^42^ Similarly, to increase feature stability and reduce noise, gray-level discretization was performed with 32 gray levels.^28^^,^^42^^,^^43^ This number of gray levels was chosen based on research extracting features from liver tumor and muscle, but the authors noted that a moderately sized value of gray-level discretization may be applicable to broader radiomic tasks.^28^ Future work should consider the optimal discretization employed for this specific work.

After the feature extraction, the classification task was performed through rigorous methodology, employing different machine learning models and cross-validation strategies. It is important to note that different models were evaluated to assess the feasibility of this classification task. Further, different models were employed to consider how the different underlying assumptions and parameters of the different models may impact performance. A comparison across the models was also beneficial to ensure that no one model was overfitted on the data, resulting in dubiously high AUC values.

LOOCV is known to be a nearly unbiased procedure as the difference in size between the training set in each iteration and the entire dataset is small. There is much discussion about its variability and, more generally, the variance of k-fold cross-validation with different sizes of k. Although Efron^44^ was one of the first to postulate LOOCV to be unbiased but with high variance, that has since been brought into question.^45^ Bengio and Grandvalet^46^ have shown that no unbiased estimators of the variance of k-fold cross-validation exist. The authors go on to discuss that the variability of LOOCV is impacted by two conditions: (1) if the cross-validation is averaging independent estimates, then LOOCV would return lower variance because of similar reasoning to the low bias as mentioned previously, or (2) if training sets are highly correlated, then LOOCV results in high variance. Overall, LOOCV was chosen *a priori* because of the small dataset size; however, the current study also investigated 5- and 10-fold cross-validation for this small dataset to better understand the potential impact of this method in a more practical scenario. Future work will investigate independent test sets.

As presented in Fig. 4(a) and Table 6, the largest AUC value (0.69 [0.60, 0.77]) was achieved using a decision tree classifier when selecting the top four features during LOOCV. The selection of four features was based on preliminary analysis that resulted in moderate performance for classification. However, the AUC value obtained with a selection of four features failed to achieve a significant difference when comparing the AUC value achieved by the SGD classifier and selecting the top 10 features. The 10-fold and 5-fold cross-validation schemes were also implemented to assess the bias and variance of the *BAP1* classification task. There was slightly different performance across the different folds of the various cross-validation methods and different numbers of features selected (Table 6).

The most-selected feature obtained using the methodology explained in Sec. 2.6 was the GLCM cluster prominence calculated after the application of an LoG filter with a radius of 1.5 mm. GLCM cluster prominence captures “a measure of the skewness and asymmetry of the GLCM,” whereby larger values indicate asymmetry about the mean and smaller values indicate a peak near the mean value and less variation about the mean.^31^ The LoG filter first applies a Gaussian kernel to an image, which blurs the image, followed by a convolution with a Laplacian filter (the second derivative of the Gaussian kernel), which enhances the edges in the image. This filter application demonstrated that blurring and enhancing the edges of the ROIs resulted in an appreciable difference between *BAP1+* and *BAP1−* patients that was reflected in the values of the GLCM cluster prominence feature. The other top features (Table 5) were either of the GLCM or GLSZM class, both capturing second-order gray-level information about an image. In addition, all four features were selected after the application of a filter, three of which were the LoG. It is noteworthy to mention that the only other study that performed radiomics for the *BAP1* classification task reported the relevance of the GLCM cluster prominence feature, as well as the usefulness of other second-order features for classification.^41^ Further, the authors found that LoG features were the most stable when extracted from 3D segmentations. Therefore, the findings in this current study support their results.

A comparison between the CNN segmentations and the human-adjusted segmentations was conducted to evaluate the impact human-modified contours had on the classification performance. There was a statistically significant difference between the AUC value obtained from the modified segmentations and the AUC value from the unmodified CNN segmentations. This demonstrated that although this work is the first to combine deep learning for the segmentation task (which substantially reduces the time spent by a radiologist to delineate the tumor), human input was still required to ensure proper capture of the tumor. The increased accuracy of tumor delineations resulted in the moderate performance achieved in classifying *BAP1+* from *BAP1−* patients.

While this study yielded promising initial results, there are potential future directions in addition to the aforementioned discussion. First, acquiring segmentations on more sections for 3D texture analysis could result in stronger predictive performance by the classifiers as has been reported.^41^ Second, the stability of the selected features could be assessed through various measures. For example, the concordance correlation coefficient could be used to reduce the number of features based on how well-extracted feature values agree before and after image perturbation operations, i.e., rotation or erosion.^47^ An initial exploration of stability of features on this dataset has shown that larger chains of perturbations including rotation and contour randomization produced the most stable and robust feature sets.^48^^,^^49^ Third, CT images from the entire history of the patients were visually assessed to identify the scan displaying the largest tumor bulk. Therefore, the selection of scans did not control for treatment time point, which could have inherently biased the results, as some of the analyzed scans were acquired either during pretreatment or during treatment; the treatment could have potentially affected image features that were extracted if the tissue presented differently. Some scans also were acquired after talc pleurodesis, which could have had an impact on the tumor tissue in a manner similar to that of treatment. Similarly, selecting the section from each scan with the largest visible tumor could have potentially biased the results. As some of these scans had been acquired during the course of treatment, the largest tumor could have been more resilient to the treatment, and the texture features may have captured that resilience as opposed to the mutation status. Future work will expand on this pilot study and select patient scans with stricter criteria, minimizing confounding variables in the curation process. Lastly, although this work demonstrated the ability to accurately detect somatic *BAP1* mutations, the approach will be extended to detect germline *BAP1* mutations in the future.

## 5 Conclusion

The potential of radiomics for identifying *BAP1* mutations from the CT scans of PM patients was demonstrated; 2D features extracted from tumor segmentations yielded an AUC value of 0.69 [0.60, 0.77] when using a decision tree classifier. The novel use of radiomics, machine learning, and deep learning techniques in the task of differentiating between *BAP1*-mutated and wild-type tumors yielded promising results, surpassing previously reported AUC values. Although this study showed encouraging outcomes, some future directions are proposed, such as 3D texture analysis, different classification schemes, and assessment of germline mutations.

## Data Availability

A collection of data presented in this article and the code described in this paper is available upon request.
